# A Handbook of Therapeutics

**Published:** 1889-09

**Authors:** 


*A Handbook of Therapeutics.* By SIDNEY RINGER, M.D., F.R.S. Twelfth Edition. London: H. K. LEWIS. 1888.

Editions of Ringer's *Therapeutics* follow one another with such rapidity, that it has been said that the work might be looked upon as a medical periodical. This surely is high testimony of its value. But we must confess to having often found Dr. Ringer's book a disappointing one. Not that any complaint can be made about deficient quantity of information: on the contrary, it contains too much, for the enquirer gets perplexed with the multiplicity of uses to which each drug can be put. The whole book appears to us to lack system; it seems like a mass of notes taken at odd times put together without a proper attempt at selection and method. For instance, distressed at the failure of ordinary remedies to check excessive night-sweats in a case of phthisis, we turned to this edition of Ringer to see if anything unfamiliar had been recently found successful, and discovered in the Index that hyoscine "checks the sweating of phthisis," a statement that is repeated at p. 489; but on the same page it is said that "it does not apparently check perspiration." A contradiction like this does injustice to the general merits of the work. The name of the drug is spelled in three different ways.

Dr. Ringer's "Index to Diseases," if it did not contain a grave element of danger in being likely to induce perfunctory treatment, would be amusing. Whilst there is, of course, a large selection of drugs, not only for such a disease as "Confinement," but for many other common complaints, Dr. Ringer has but one suggestion to offer either for "Catch in the Breath" or for "Barrenness;" whilst "Crick in the

Neck" appearing in the list, isolated and without comment, seems to be altogether beyond the therapeutic resources of the present time, to which Dr. Ringer has so materially added by his original and laborious investigations.

